# Spatial modeling, prediction and seasonal variation of malaria in northwest Ethiopia

**DOI:** 10.1186/s13104-019-4305-1

**Published:** 2019-05-14

**Authors:** Asefa Adimasu Taddese, Adhanom Gebreegziabher Baraki, Kassahun Alemu Gelaye

**Affiliations:** Department of Epidemiology and Biostatistics, Institute of Public Health College of Medicine and Health Sciences, University of Gondar, Gondar, Ethiopia

**Keywords:** Spatial analysis, Interpolation, Bayesian kriging, Clustering, Geostatistics modeling

## Abstract

**Objectives:**

The aim of this study was to determine the spatial modeling, seasonal variation of malaria and making prediction map of malaria in northwest Ethiopia.

**Results:**

The overall average cumulative annual malaria incidence during the study period was 30 per 100 populations at risk. The highest proportion (29.2%) was observed from June 2015 to October 2016. In temporal analysis of clusters, the epidemic was observed from 2015/7/1 to 2016/12/31 throughout the study period in all districts. Hotspot areas with high clusters (p < 0.001) were observed in Metema district it accounts 18.6% of the total malaria cases. An area of high median predicted incidence proportion (> 50%) was seen in the southwest part of the region. Most of the northern part of the study area was predicted to have a low median incidence proportion (< 10%).

**Electronic supplementary material:**

The online version of this article (10.1186/s13104-019-4305-1) contains supplementary material, which is available to authorized users.

## Introduction

Malaria is a mosquito-borne infectious disease of humans caused by the genus Plasmodium, which are introduced into the circulatory system by the bite of an infected female anopheles mosquito [1]. It is one of the major public health challenges undermining development in the poorest countries [1–4].

Malaria is largely seasonal in Ethiopia, with the major incident occurring during the rainy.

Season from April to October [5]. In Ethiopia 75% of the country is malarious with about 60% of the total population living in areas at risk of malaria. That is, 50.6 million people are at risk from malaria, and four to five million people are affected by malaria annually [1, 6].

The study area is one of the lowland malarious regions in Ethiopia. The dominant species of malaria in the region are both *P. falciparum* and *P. vivax* [7]. It accounts 19% of the national malaria burden [7, 8]. Currently, the study area accounts 31% (1.3 million cases) of Ethiopia’s malaria burden [9].

Malaria mosquitos are aggregated over large areas and time periods. There are few studies examining the extent and drivers of local variation in malaria exposure.

In geographical location, close proximity share common exposures which influence the disease outcome. Ignoring the potential spatial correlation in neighboring areas due to common exposure could result in incorrect model estimates. A little research was conducted by considering spatial correlation. But, using a geostatistical modeling with Bayesian framework takes into account spatial clustering by introducing location-specific random effect parameters in the covariance matrix of a function of distance between locations.

The findings of this study can be used to increase the evidence for targeting control measures and will contribute to the development of models capable of predicting future malaria scenarios.

## Main text

### Methods

A repeated cross-sectional study was conducted from 2014 to 2017 in the north Gondar zone, northwest Ethiopia, which is located in the north western part of Ethiopia (Additional file 1).

Topographically, the study area contains lowlands, 552, to highlands, 4620 m above sea level [8]. The study area has had 28.7 °C; 14.1 °C and 0% to 87.5% mean maximum and minimum temperature and relative humidity respectively. The study area also had and two rainy seasons: the main one is from June to September, followed by a shorter one from March to May. The dry season ranges from October to February [9].

Malaria data were obtained from monthly reported surveillance forms of north Gondar zone health offices between early 2014 and late 2017. The data were collected from the study population, that is patients who visit health institutions in the study area and were aggregated at woreda levels. The spatial coordinates (the latitudes and longitudes) for each woreda were obtained from the Ethiopian demographic and health survey (EDHS) GPS data reference.

For clustering and spatial pattern was detected by using ArcGIS and Sat Scan™ software, version 9.1 [10] using the Kulldorf method.

For computing hypothesis (Ho) test and the presence spatial autocorrelation we used global and local Moran’s I test statistic [11]. Significant clustering, variable or dissimilar patterns and random patterns were declared when the mean Moran’s I values are positive, negative and zero respectively. Z score values were used to see local clustering of malaria cases. Z score values above 1.96 and below negative 1.96 were used to show hotspot and coldspot areas respectively and any value between the two shows random distribution of cases. Interpolation was done using Empirical Bayesian kriging for predictions and smoothing.

### Result

About 916,204 malaria cases which were reported from the study period, 69.8% were plasmodium falciparum, 26.8% plasmodium vivax and 2.2% of the case were mixed infected. 71.78% of total malaria cases were observed in adults aged above 15 years old and 8.5% were childrens under 15 years old.

**Fig. 1:**
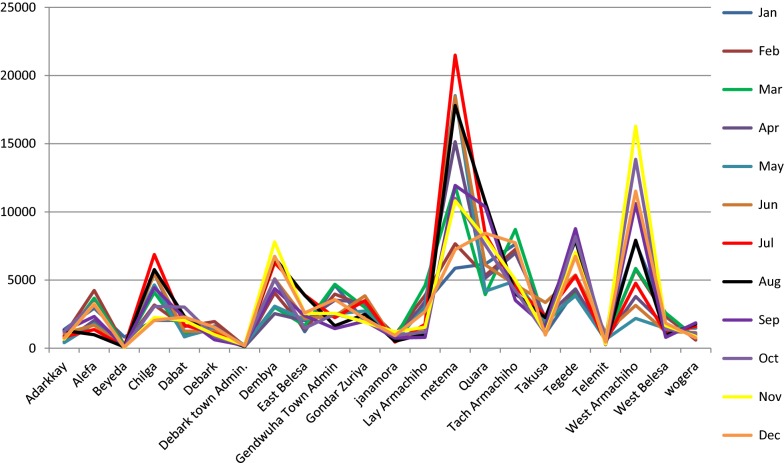
Monthly spatial distribution of malaria at woreda level in North Gondar Zone, northwest Ethiopia from 2014 to 2017

We have identified two major malaria transmission periods, the major transmission time starts from mid-April with highest occurrence in June and July this falls to its lowest in December. The second high transmission was from October to November (Fig. 1).

Even though the cumulative annual malaria incidence was 30 per 100 populations at risk, it has showed a significant variation across districts and months. The highest incidence (44.8%) occurred in Metema district from July to November 2016. Whereas, lowest incidence (5%) occurred in Debark Town administration district from late November to the end of December 2014. From the total malaria cases reported the largest percentage (29.2%) occurred from June 2015 to October 2016 and the lowest (19.1%) observed in 2017 (Additional file 2).

Spatital clustering of cases was detected; Gendawuha Town Administration and Metema districts being the most likely clusters (LLR = 184,968.375, p < 0.001). Secondary cluster was West Armachiho district (LLR = 8936.64, p < 0.001) and the third cluster was Quara district (LLR = 36,249.66, p < 0.001) (Table 1).

**Table 1:** Significant high rate spatial cluster of malaria in North Gondar Zone; northwest Ethiopia from 2014 to 2017

Cluster	District	Coordinates/radius	Observed case	Expected case	RR	LLR
1	Metema	12.95N, 36.15E)/1 km	19,355	35,811	6.7	184,968.3*
1	Gendawuha	12.95N, 36.15E)/1 km	19,355	35,811	6.7	184,968.3*
2	West Armachiho	(13.22N, 36.43E)/1 km	93,555	8936.6	11.6	139,430.3*
3	Quara	12.50N, 35.75E)/1 km	84,478	29,247.4	3.09	36,249.66*
4	Tegede	(13.50N, 37.10E)/1 km	69,808	23,072.1	3.20	31,877.61*
5	Tach Armachiho	(13.22N, 37.14E)/1 km	69,163	28,193.9	2.58	22,118.95*

* p value ≤ 0.001

High rates of spatio-temporal malaria clusters were identified in Dembia district (LLR = 1628.51, p < 0.001) from 2014/01/01 to 2017/12/31. Secondary clusters were identified in the East Belesa district (LLR = 1469.21, p < 0.001) from 2014/1/1 to 2017/12/31 (Additional file 3).

Purely temporal high malaria clusters were observed from 2015/7/1 to 2016/12/31 (LLR = 66, 53.42, p < 0.001) throughout the study period across all districts.

**Fig. 2:**
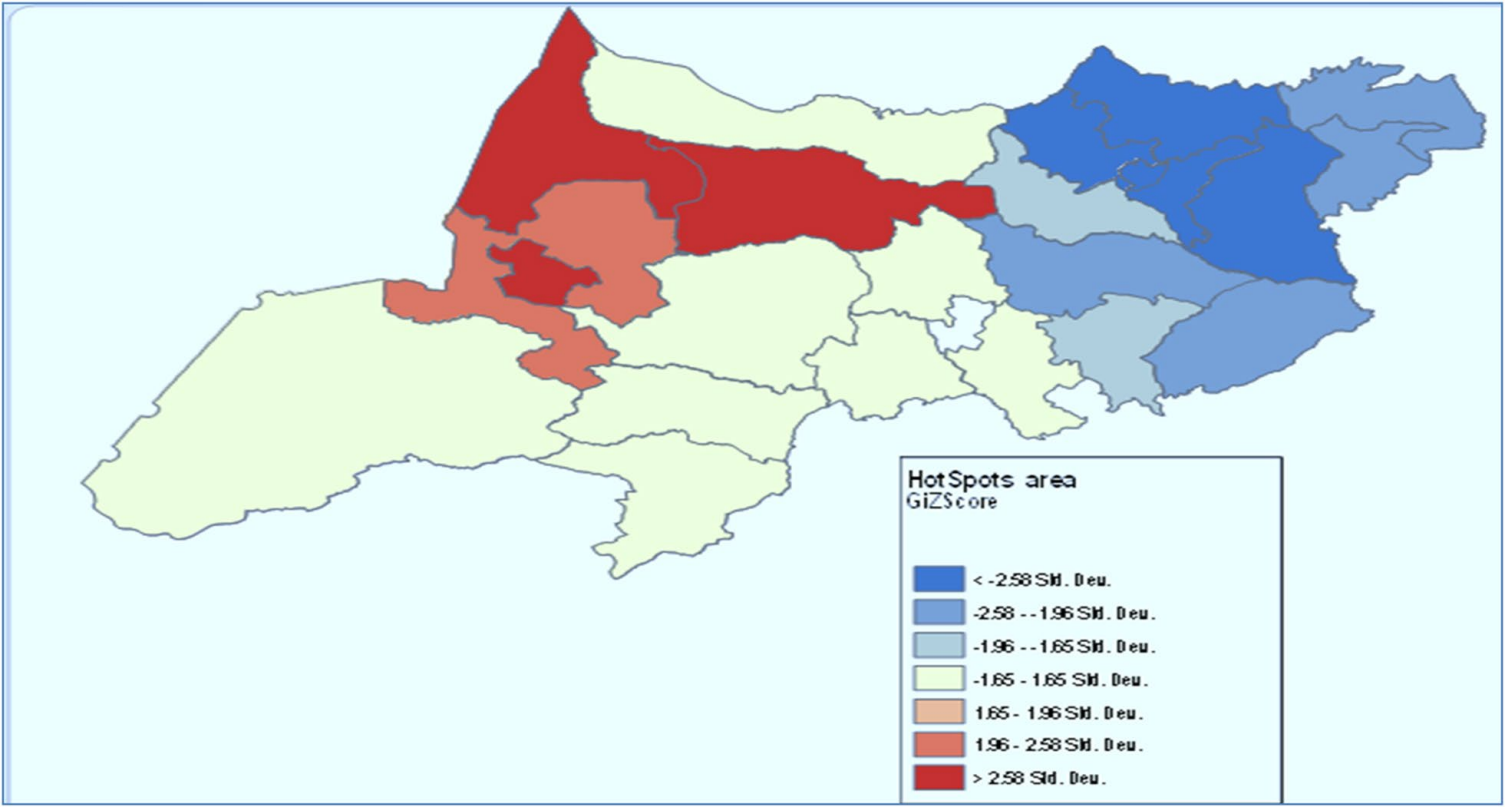
Hot spot identification of malaria prevalence in north Gondar zone, northwest Ethiopia between 2014 and 2017

Hot spot areas with high cluster of malaria transmission and cold spot areas with low level clusters were identified. Hotspot areas with high clusters (p < 0.001) were seen in Metema district it covers 18.6% of the total malaria cases (Fig. 2).

Districts that had lower malaria incidence proportion in the region are indicated by high bright green color on the map and they were clustered around the North and east part of the study area, while the high malaria incidence proportion are indicated by high dark and less dark red color which are located in the Western part of the study area.

The maximum peak, where spatial clustering highly pronounced is at a distance of 135.778 kms with corresponding Z score of 9.81 (p-value < 0.00). This distance band is used for the analysis of hot spot and cold spot clusters (Additional file 4).

The global Moran’s index statistic for the malaria incidence per 100,000 population was 0.31 (p-value = 0.007), indicating the presence of significant positive spatial autocorrelation over the whole study area.

In this analysis the z-score with corresponding p-values showed that there is statistically significant autocorrelation at 0.1, 0.05 and 0.01 level of significance (Additional file 5).

An area of high median predicted incidence proportion (> 50%) was seen in the southwest part of the region. Most of the northern part of the study area was predicted to have a low median incidence proportion (< 10%). The area of highest predicted incidence proportion within the study area corresponds with the majority of high incident districts. The black ramp color indicates the predicted malaria high risk areas and red ramp color indicates less risk areas of malaria (Additional file 6).

### Discussion

In study area, the overall average cumulative malaria incidence proportion was 30%. Thus, results were higher than studies conducted in other parts of Ethiopia (9.7%) [12], (4.3%) [7], (6.8%) [6], (10.4–13.5%), (7.6–14.1%), (5.4%) [13] and (4.5%) [14]. But, lower than studies conducted in 2011 (43%) and in 2014 (33%) in Tigray regional state [15]. Higher malaria incidence in the study area might be the result different climatic variables [16–20]; the study area has a long rainy season and temperature variability [21].

The dominated species plasmodium falciparum were covered 69.8% of the total malaria cases. Other studies also agreed that plasmodium falciparum were the dominant species in the northwest parts of Ethiopia [15, 22] and both *Plasmodium falciparum* and *Plasmodium vivax* is common in Amhara region [7]. The reason may relate to temperature; temperatures more than 18 °C for p. Falciparum and more than 15 °C for vivax are suitable for the growth of these two species. Obviously, in the most part of the country, especially in northwest Ethiopia, temperature is greater than the specified minimum scale [23]. The above idea was supported by researchers in Tanzania [24].

The 71.8% of malaria victims were adults aged above 50 years. This is in agreement with other studies [12, 14]. However, other studies reversely stated that malaria infection decreased with increasing age group (70); it reasoned out higher body temperature individuals had more susceptible to malaria, which are incapable of clearing parasites more effectively as adults. Even though, the study area is an active investment and agricultural hot spot areas in Ethiopia whereby there are frequent adult immigration from another part of the country.

The 29.2% cases from the total cases were recorded from July 2015 to December 2016. It is supported by other studies in Ethiopia [9, 15, 25], because of high malaria burden in high-risk geographic areas [26].

The major transmission period were observed from the beginning of mid-April and peaked in June and July, declining at the end of December. The second peak of was observed between October and November. Other studies agreed that in Ethiopia season there are two peak malaria transmission periods, but the occurrence months were varied [27, 28]. However, whatever the starting point all agreed that malaria transmission were high at the first wet season (summer rains) while the latter followed the end of the wet season [28].

The spatio-temporal models indicated that the most likely cluster was located in Dembia district and in East Belesa. All these identified clusters are closely related to a specific geographical area and share similar geographical parameters, such as altitude and weather conditions.

Spatial clusters were detected in Metema, Tach Armachiho and Gendawuha town administration districts, all of which are located in the Ethiopia–Sudan border regions of the country. These districts are located far from referral hospitals and contain a high number of seasonal migrants due to the presence of agricultural investments and malaria is poorly controlled in these travelers and hard to reach populations. This highlights the risk of cross-border transmission of malaria in Ethiopia and Sudan, particularly in predominantly male migrant populations.

The highest predicted malaria incidence was observed in the region of Dembia, Takusa, Metema district (North West part of the zone) this region can be attributed to sesame (local name “selit”) growing which is a predominant economic activity. The sesame paddies are suitable habitats for malaria vector breeding. In southeastern region and central North part of malaria is low largely due to its location in the highlands [29].

### Conclusions

Malaria is still a major public health problem in the study area with an overall average cumulative annual incidence of 30 per 100 populations at risk. This malaria incidence proportion in the region was higher than the country statistics. Malaria incidence has also showed a significant spatio-temporal clustering. Most of the northern part of the study area is predicted to have a low median incidence.

## Limitations

As the data were obtained from passive surveillance system all clinical records did not fully capture the level of malaria transmission in the districts because some people do not report to the formal clinics, either using traditional therapists or self-diagnose and purchase their own drugs.

## Additional files

**Additional file 1.** Location of Ethiopia, Amhara National Regional State and the study area; Source of shape file:Amhara region central statistics agency 2017 shape file.

**Additional file 2.** Monthly and yearly variation of malaria transmission in North Gondar zone, northwest Ethiopia.

**Additional file 3.** Significant high rate Spatio-temporal malaria clusters in North Gondar Zone; northwest Ethiopia from 2014 to 2017.

**Additional file 4.** Spatial autocorrelation based on feature locations and attribute values using the Global Moran’s I statistic.

**Additional file 5.** Spatial Incremental autocorrelation of malaria by distance in North Gondar Zone; northwest Ethiopia from 2014 to 2017.

**Additional file 6.** Interpolated maps of predicted malaria incidence in north Gondar zone, northwest Ethiopia between 2014 and 2017.

## Data Availability

The data upon which the result based could be accessed based on a reasonable request to the corresponding author

## Abbreviations

CDC: Communicable Disease Control
CSA: Central Statistical Agency
FMOH: Federal Minister of Health
GIS: geographical information system
GPS: geographical positioning system
LLR: log-likelihood ratio
RR: relative risk
WHO: World Health Organization
