# Supplementary material for: Spatial modeling, prediction and seasonal variation of malaria in northwest Ethiopia
Source: BMC Res Notes. 2019 May 14;12:273. doi: 10.1186/s13104-019-4305-1 (PMC6518452; doi:10.1186/s13104-019-4305-1)

Additional file 1: Location of Ethiopia, Amhara National Regional State and the study area; Source of shape file:Amhara region central statistics agency 2017 shape file


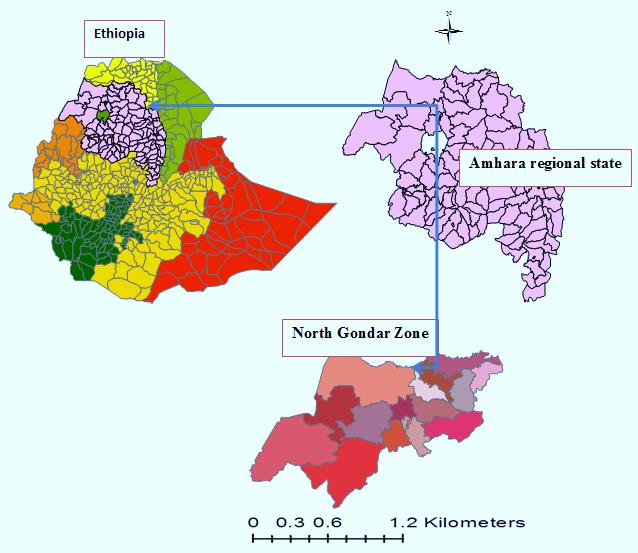

Supplement: Supplementary file 1 — Additional file 1. Location of Ethiopia, Amhara National Regional State and the study area; Source of shape file:Amhara region central statistics agency 2017 shape file. [file 13104_2019_4305_MOESM1_ESM.docx]
