# Supplementary material for: Spatial modeling, prediction and seasonal variation of malaria in northwest Ethiopia
Source: BMC Res Notes. 2019 May 14;12:273. doi: 10.1186/s13104-019-4305-1 (PMC6518452; doi:10.1186/s13104-019-4305-1)
Supplement: Supplementary file 2 — Additional file 2. Monthly and yearly variation of malaria transmission in North Gondar zone, northwest Ethiopia. [file 13104_2019_4305_MOESM2_ESM.docx]

Additional file 2: Monthly and yearly variation of malaria transmission in North Gondar zone, Northwest Ethiopia
