# Supplementary material for: Spatial modeling, prediction and seasonal variation of malaria in northwest Ethiopia
Source: BMC Res Notes. 2019 May 14;12:273. doi: 10.1186/s13104-019-4305-1 (PMC6518452; doi:10.1186/s13104-019-4305-1)
Supplement: Supplementary file 3 — Additional file 3. Significant high rate Spatio-temporal malaria clusters in North Gondar Zone; northwest Ethiopia from 2014 to 2017. [file 13104_2019_4305_MOESM3_ESM.docx]

**Additional file 3: Significant high rate Spatio-temporal malaria clusters in North Gondar Zone; northwest Ethiopia from 2014-2017.**

| Cluster | District | Coordinate/ Radius | Time frame | LLR | P_ Value | Observed Cases | Expected Cases | RR |
| --- | --- | --- | --- | --- | --- | --- | --- | --- |
| 1 | Dembia | 12.24N ,37.29E/1km | 2014/1/1-2017/12/31 | 1628.5 | 0.00 | 59744 | 84863.5 | 0.68 |
| 2 | East Belesa | 12.41N, 38.09E/1km | 2014/1/1-2017/12/31 | 1469.2 | 0.00 | 27373 | 30928.0 | 0.88 |
| 3 | Tach Armachiho | 13.22N,37.15E/1km | 2014/1/1-2017/12/31 | 1050.5 | 0.00 | 69163 | 28193.9 | 2.58 |
| 4 | Dabat | 12.98N,37.75E/1km | 2014/1/1-2017/12/31 | 455.23 | 0.00 | 21262 | 34325.4 | 0.61 |
| 5 | Beyeda | 13.30N,38.15E/1km | 2014/1/1-2017/12/31 | 134.55 | 0.00 | 2247 | 28593.9 | 0.08 |
| 6 | Alefa | 11.93N, 36.87E/1km | 2014/1/1-2017/12/31 | 78.68 | 0.00 | 30916 | 53183.5 | 0.57 |
| 7 | Adarkkay | 13.45N,38.06E/1km | 2014/1/1-2017/12/31 | 67.38 | 0.00 | 11002 | 29504.0 | 0.36 |
| 8 | Takusa | 12.19N, 37.06E/1km | 2014/1/1-2017/12/31 | 23.40 | 0.00 | 21790 | 40160.5 | 0.53 |
| 9 | Debark | 13.14N,37.90E/1km | 2014/1/1-2017/12/31 | 9.28 | 0.00 | 15342 | 37503.8 | 0.40 |
| 10 | Debark Town | 13.32N, 37.96E/1km | 2014/1/1-2017/12/31 | 7.06 | 0.00 | 2090 | 11727.2 | 0.18 |
