# Supplementary material for: Spatial modeling, prediction and seasonal variation of malaria in northwest Ethiopia
Source: BMC Res Notes. 2019 May 14;12:273. doi: 10.1186/s13104-019-4305-1 (PMC6518452; doi:10.1186/s13104-019-4305-1)
Supplement: Supplementary file 4 — Additional file 4. Spatial autocorrelation based on feature locations and attribute values using the Global Moran’s I statistic. [file 13104_2019_4305_MOESM4_ESM.docx]

| Moran's Index: 0.311354  Expected Index: - 0.047619  Variance: 0.018127  z-score: 2.666581 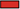  p-value: 0.007663  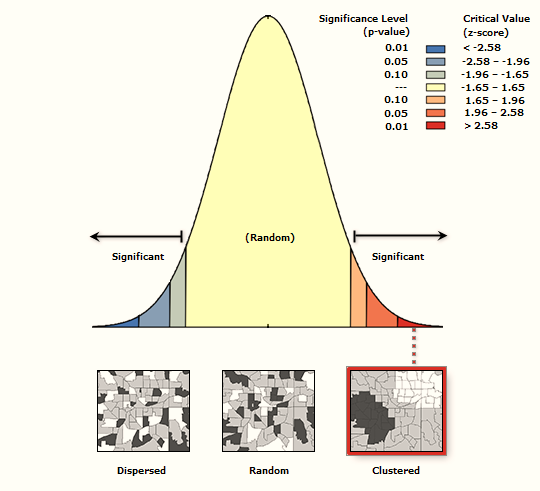 |
| --- |

Additional file 4: Spatial autocorrelation based on feature locations and attribute values using the Global Moran's I statistic.
