# Supplementary material for: Spatial modeling, prediction and seasonal variation of malaria in northwest Ethiopia
Source: BMC Res Notes. 2019 May 14;12:273. doi: 10.1186/s13104-019-4305-1 (PMC6518452; doi:10.1186/s13104-019-4305-1)
Supplement: Supplementary file 5 — Additional file 5. Spatial Incremental autocorrelation of malaria by distance in North Gondar Zone; northwest Ethiopia from 2014 to 2017. [file 13104_2019_4305_MOESM5_ESM.docx]

**Additional file 5: - Spatial Incremental autocorrelation by distance of malaria in North Gondar Zone; Northwest Ethiopia from 2014-2017.**

| Distance (Meter) | Moran’s I | Expected I | Variance | Z-score | P-value |
| --- | --- | --- | --- | --- | --- |
| 95778.00 | 0.73 | -0.05 | 0.01 | 8.89 | 0.00 |
| 115778.00 | 0.56 | -0.05 | 0.00 | 9.26 | 0.00 |
| 135778.00 | 0.46 | -0.05 | 0.00 | 9.81 | 0.00 |
| 155778.00 | 0.30 | -0.05 | 0.00 | 8.50 | 0.00 |
| 175778.00 | 0.15 | -0.05 | 0.00 | 6.54 | 0.00 |
| 195778.00 | 0.07 | -0.05 | 0.00 | 5.02 | 0.00 |
| 215778.00 | 0.04 | -0.05 | 0.00 | 4.31 | 0.00 |
| 235778.00 | -0.01 | -0.05 | 0.00 | 2.69 | 0.01 |
| 255778.00 | -0.04 | -0.05 | 0.00 | 1.39 | 0.17 |
| 275778.00 | -0.04 | -0.05 | 0.00 | 0.83 | 0.40 |

First Peak (Distance, Value): 135778.00, 9.811839

Max Peak (Distance, Value): 135778.00, 9.811839
