# Supplementary material for: Spatial modeling, prediction and seasonal variation of malaria in northwest Ethiopia
Source: BMC Res Notes. 2019 May 14;12:273. doi: 10.1186/s13104-019-4305-1 (PMC6518452; doi:10.1186/s13104-019-4305-1)

Addition file 6: Interpolated maps of predicted malaria incidence in north Gondar zone, northwest Ethiopia between 2014 and 2017


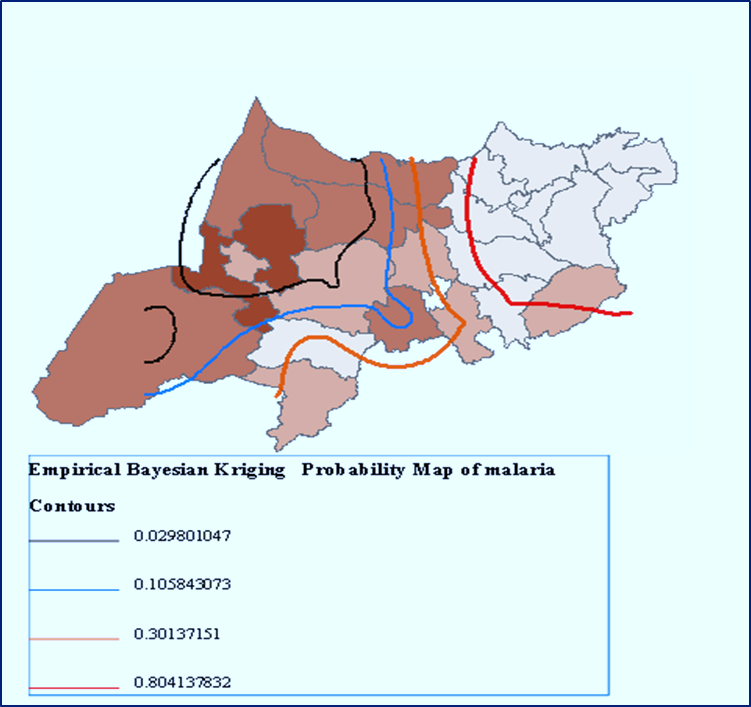

Supplement: Supplementary file 6 — Additional file 6. Interpolated maps of predicted malaria incidence in north Gondar zone, northwest Ethiopia between 2014 and 2017. [file 13104_2019_4305_MOESM6_ESM.docx]
